# Impact of Smoking and Deprivation in Patients Admitted With Severe COVID-19 Infection to ICU: A Retrospective Cohort Study

**DOI:** 10.1155/ipid/2029135

**Published:** 2025-11-03

**Authors:** James Ainsworth, Suresh Pillai

**Affiliations:** Ed Major Intensive Care Unit, Morriston Hospital, Heol Maes Eglwys, Treforys, Cwmrhydyceirw, Swansea SA6 6NL, Wales, UK

## Abstract

**Introduction:**

Smoking is known to be harmful to health, contributing to increased severity in respiratory infections such as influenza. The impact in COVID-19 remains controversial. Social deprivation and inequality are associated with worse outcomes in infectious disease and may impact on mortality in COVID-19.

**Methods:**

A retrospective cohort study in a tertiary hospital general adult Intensive Care Unit (ICU). 174 patients with COVID-19 confirmed by polymerase chain reaction (PCR) testing between March 21st 2020 and May 15th 2021 included. Data collection was by retrospective review of clinical notes. Patients were grouped into three categories: current smokers, former smokers (ex-smokers) and never smokers. Statistical analysis carried out on IBM Statistical Package for Social Sciences (SPSS) for Windows, version 28.0 (Armonk, NY:IBM Corp). Values reported as means, standard deviation (SD), median and interquartile ranges (IQR). Comparisons with the Mann–Whitney *U* test for median and IQR and two sample *t*-test for mean and SD. Survival analysis was performed using Kaplan–Meier.

**Results:**

Of 174 patients, 11 were current smokers (6.3%), 51 were former smokers (29%) and 112 were never smokers (64%). Current smokers were younger and frailer, with longer hospital and ICU length of stay (ICULOS). Welsh Index of Multiple Deprivation (WIMD) scores were collected using UK postcodes and ranked 1909 areas, showing current smokers from the most deprived areas. Kaplan–Meier survival analysis showed current smokers are less likely to survive.

**Discussion:**

Despite 6.3% prevalence of smoking in patients with severe COVID-19 in the ICU, smokers had increased hospital and ICU LOS with reduced survival. The relationship between COVID-19 and deprivation is multifaceted and complex and may relate to demographic, socioeconomic and environmental factors; health-related risk factors; and practices. From our data we can see that current smokers admitted to ICU with severe COVID-19 were from the most deprived areas in Wales, with a higher mortality risk.

## 1. Introduction

Smoking is known to be harmful to health, with detrimental effects on both the respiratory and cardiovascular systems. It is linked with numerous diseases and health conditions, including many cancers, and contributes to a significant disease burden and mortality worldwide. Smoking is the main cause of chronic obstructive pulmonary disease (COPD); 80%–90% of COPD patients being smokers or former smokers [1]. According to the World Health Organisation (WHO), smoking kills over 8 million people each year, 1.2 million of whom as a result of exposure to second hand smoke. Smoking is common, with an estimated 1.3 billion tobacco users worldwide, the majority living in low- and middle-income countries [2, 3]. Smoking impacts the immune system and response to infection, causing greater vulnerability to infections and contributing to severity of disease in respiratory infections such as influenza, tuberculosis and community acquired pneumonia [3]. “Current smokers are five times more likely to develop influenza infection than nonsmokers” [4, 5].

Smokers would be expected to be at increased risk from COVID-19; evidence and opinions however remain controversial. Many studies suggest a low prevalence of smoking, frequently below 10%, in hospitalised patients with COVID-19 [4, 6–11]. A meta-analysis in China which reviewed a total of 7168 patients found a smoking prevalence of under 7%, compared with an expected pooled prevalence of smoking of 24.1% [12]. Similar results were found in Italy which did not have any current smokers in their cohort of 132 patients, compared with a local population prevalence of 22.7% smokers [13], in France with a prevalence of 4.4% of hospitalised COVID-19 patients being smokers [14] and in Iran with only 3.06% of patients in one study exploring factors influencing COVID-19 severity being smokers [15]. An observational case control study at a single trust in London carried out between March and August 2020 comparing smoking hospitalisations with COVID-19 and other respiratory viruses pre-COVID-19 suggested current smokers were less likely to be admitted to hospital with COVID-19 compared with other respiratory viruses the previous year [16]. A large cohort study in England with 7,869,534 people, with the aim of being representative of the English population, and included a review of patient demographics and smoking status, concluded that current smokers were at lower risk of severe COVID-19 infection. All-cause mortality however remained higher in current smokers compared with nonsmokers, despite a possible lower mortality associated with COVID-19 [17].

On the other hand, many studies suggest an increased risk of severe disease in smokers admitted to hospital with COVID-19, with worse outcomes, increased ICU admission and higher mortality [18], for example, the article “Effect of chronic obstructive pulmonary disease and smoking on the outcome of COVID-19,” a meta-analysis investigating the association between COPD and smoking and outcomes in patients with COVID-19, specifically disease severity, ICU admission and mortality. Data were collected from a total of 21 studies, amounting to a total of 4603 patients. This review suggests that COPD and smoking are associated with worse outcomes in those admitted with COVID-19, including increased mortality and ICU admissions [18]. The Centres for Disease Control (CDC) includes smoking on a list of conditions demonstrating higher risk [19].

Health inequity and its interaction with smoking and COVID-19 outcomes are important aspects of the pandemic's impact. Structural and social determinants influence health behaviours, including smoking, and contribute to variations in disease prognosis. In this study, we aimed not only to assess the impact of smoking on outcomes in patients with severe COVID-19 admitted to ICU but also to explore how deprivation and socioeconomic factors intersect with smoking behaviours, potentially influencing these outcomes in a context specific manner.

Socioeconomic status and geographic inequality appear to impact the associated risk with COVID-19, areas with lower socioeconomic status or deprivation demonstrating higher mortality than more affluent areas. In some circumstances, this may be related to higher minority ethnic populations, which may confer a higher risk [20]. Some studies found that more socially disadvantages areas have a higher COVID-19-related mortality. Northern regions in England are relatively more deprived and have shown higher death rates related to COVID-19, with a two- to threefold elevated risk in the most disadvantaged 20% of the UK [20, 21]. This has not been consistent across all studies. Other geographic inequalities associated with increased rates of infection have been noted previously with other infectious pandemics or epidemics, such as H1N1 influenza in 2009, and outbreaks of Ebola in the Congo [20]. The pandemic itself may have actually contributed to changes in health behaviour, with significantly increased rates of smoking, e-cigarette use and alcohol consumption, in addition to increased sedentary behaviour and diet changes [22, 23]. It is useful to understand the effects of social deprivation and specific lifestyle factors; whether they influence the risk of infection and outcomes in COVID-19 in a similar way to that which is seen with other infectious diseases and other notable diseases such as cardiovascular disease, which is also more prominent in lower socioeconomic regions and associated with lifestyle factors such as smoking [21]. This study investigates smoking status and the impact of smoking on patients admitted to intensive care with COVID-19 during the first and second wave of the pandemic. Baseline patient characteristics are reviewed such as variability between smokers, former smokers and nonsmokers. Data on socioeconomic status and deprivation of all patients are collected, using Welsh Index of Multiple Deprivation (WIMD) scores for current, former and never smokers, based on the UK postcodes.

## 2. Methods

This is a retrospective study that was carried out in a tertiary hospital general adult Intensive Care Unit (ICU). All patients who were admitted to the ICU during the first and second waves of the COVID-19 pandemic from March 2020 to May 2021 were included. The patients included were over the age of 18 and had confirmed COVID-19 by SARS-CoV-2 polymerase chain reaction (PCR) testing. Data collection was by retrospective review of the clinical notes. The study did not require an ethical review as advised by the Research and Development (R&D) department. The patients were grouped into three categories: current smokers, former smokers and never smokers. All statistical analysis was carried out on IBM Statistical Package for Social Sciences (SPSS) for Windows, version 28.0 (Armonk, NY:IBM Corp). The values are reported as means and standard deviation (SD) or median and interquartile ranges (IQR) where appropriate. Comparisons were done with Mann–Whitney *U* test for median and IQR and two sample *t*-test for mean and SD. Data were deemed significant when *p* < 0.05. Survival analysis was performed using Kaplan–Meier. This article is a subanalysis of previously published work [24].

## 3. Results

**Figure 1:**
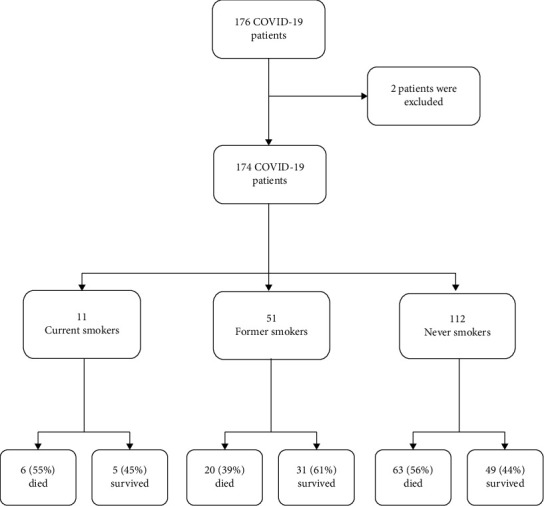
Flow diagram showing patient recruitment.

A total of 57 patients from the first wave and 119 patients from the second wave were included in the study. Out of 176 patients, 2 were excluded because of the lack of smoking history and 174 patients were included in the analysis. There were 11 current smokers, 51 former smokers (ex-smokers) and 112 never smokers (nonsmokers) (Figure 1).

The current smokers were significantly young when compared to former smokers and never smokers. There were more male patients admitted when compared to females in all groups. When compared to never smokers, current smokers were significantly frail. The hospital length of stay (HLOS) and ICU length of stay (ICULOS) were significantly high in the current smokers. Hypertension was the commonest comorbidity. As expected, COPD was more prevalent among current smokers (Table 1).

**Figure 2:**
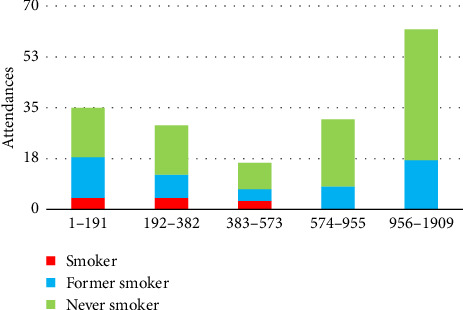
Welsh index of multiple deprivation (WIMD) scores for current, former and never smokers.

WIMD 2019 rank is based on the UK postcodes and was ranked from 1 (most deprived) to 1909 (least deprived) and was grouped into quintiles 1–191, 192–382, 383–573, 574–955 and 956–1909 (the WIMD 2019). The study showed that current smokers were from the most deprived areas (Figure 2).

**Figure 3:**
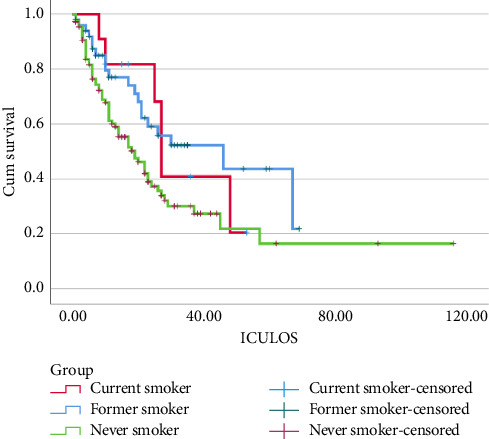
Kaplan–Meier survival analysis showed that current smokers admitted to ICU with severe COVID-19 infections are less likely to survive (log rank [mantel-cox], *p*=0.03).

The Kaplan–Meier survival analysis showed that critically unwell COVID-19 patients admitted to ICU who are current smokers are less likely to survive (Log Rank [Mantel-Cox], *p*=0.03) (Figure 3).

Due to the small sample size of current smokers (*n* = 11), further stratification by deprivation quintiles was limited, and results should be interpreted with caution. Although the WIMD data indicated that current smokers came from more deprived areas, the sample size constrained meaningful subgroup or interaction analysis. We recognise that this limits the strength of conclusions that can be drawn regarding the interaction between deprivation and smoking in this cohort.

## 4. Discussion

Despite a huge amount of research into COVID-19, the association between smoking, deprivation and COVID-19 outcomes remains complex. Older age, male sex, obesity and various comorbidities, particularly hypertension, appear to be consistent risk factors associated with admission to hospital and worse outcomes [16]. There are differing results and opinions regarding smoking however, with some studies suggesting increased risk of infection, severe disease and adverse outcomes whereas others suggest a low prevalence of smoking in patients admitted to hospital with COVID-19 and that smoking may even exert a protective effect against SARS-CoV-2 or COVID-19. Our study showed a low prevalence of current smoking (6.3%) among ICU patients with severe COVID-19, consistent with previous observations. However, smokers had increased hospital and ICULOS and reduced survival compared with nonsmokers, in keeping with findings that smoking may contribute to disease severity. Interestingly, former smokers had better outcomes than never smokers. This is unlikely to reflect a true protective effect, but rather residual confounding or the effect of chance given the small sample size.

A central focus of this study was the association between deprivation and smoking and how these social determinants may influence COVID-19 outcomes. Our data suggested that current smokers admitted to ICU were from the most deprived areas in Wales, reflecting the association between lower socioeconomic status and higher smoking prevalence. However, due to the limited sample size, we could not robustly analyse the interaction between deprivation levels and smoking on mortality, which should be explored in future research.

The association of COVID-19 mortality risk and deprivation is multifaceted and complex and may relate to a number of demographic, socioeconomic and environmental factors as well as health-related risk factors and practices, many of which may be confounding or contributing factors. Higher smoking prevalence but also increased drug and alcohol use, increased poverty and unemployment may all have an impact [20]. Health inequalities, such as reduced access or testing capabilities or quality of testing (e.g., in lower income countries or areas within developed countries), different policies for testing, or worse health seeking behaviours in smokers, may contribute to a degree of underestimation of numbers in some studies [11, 25]. Different working and living conditions and population density may also have a role in increasing transmission of transmissible diseases. When considering the epidemiology of smoking and disease, confounding factors such as lower socioeconomic status must also be considered [10, 26]. Smoking is known to be more prevalent in lower socioeconomic communities [11]. From our data, we can see that based on the patients UK postcodes and using the WIMD scoring, current smokers admitted to ICU with severe COVID-19 were from the most deprived areas in Wales and a higher mortality risk.

Confounding is a significant concern in observational studies, particularly given differences in age, frailty and COPD prevalence among groups. These factors may bias the observed association between smoking and outcomes, and our analysis did not adjust for these potential confounders. Future studies using larger datasets should employ multivariable analyses to clarify the independent effects of smoking and deprivation on COVID-19 prognosis.

### 4.1. Limitations

This study has several limitations. Firstly, it was a single-centre retrospective observational cohort study, limiting the generalisability of findings. Secondly, the small sample size, particularly the very small number of current smokers (*n* = 11), restricts statistical power and the reliability of subgroup comparisons, and thus these results should be interpreted with caution. Thirdly, there were baseline differences between groups, including age, frailty and COPD prevalence, introducing potential confounding that was not adjusted for using multivariable methods. The observational design is inherently subject to biases, including potential misclassification of smoking status and unmeasured confounding factors. Because of the small sample size, particularly for current smokers, we did not attempt multivariable adjustment, which would not be statistically robust. The associations observed may therefore be influenced by confounding factors such as age, frailty and comorbidities. Variations in ICU admission criteria during the pandemic may have contributed to the lower proportion of current smokers, as those with severe comorbidities or poor prognosis may have been less likely to be admitted to ICU. We were unable to test the proportional hazards assumption in our Kaplan–Meier analysis, and the absence of sensitivity analyses further limits the robustness of our findings. These limitations should be considered when interpreting the results, which should be viewed as exploratory and hypothesis-generating. Overall, while this small size limits definitive generalisable conclusions, this study's local deprivation–focussed ICU cohort provides meaningful context specific insight into the relationship between smoking, socioeconomic deprivation and severe COVID-19 outcomes relevant to our local ICU setting and population, highlighting health inequalities in Wales, and may be of use for local health policy and service planning. Future research should aim to use multicentre data, adjusted analyses and larger sample sizes to further understand these complex interactions and to inform targeted public health interventions.

## 5. Conclusions

Patients admitted to ICU with severe COVID-19 infection who are current smokers are less likely to survive. Current smokers are from the most deprived areas demonstrating the link between smoking and deprivation.

## Figures and Tables

**Table 1:** Baseline characteristics of the critically unwell COVID-19 patient groups.

	Current smoker (*n* = 11)	Former smoker (*n* = 51)	Never smoker (*n* = 112)
Age (years)	50 ± 8	61 ± 12^∗^	57 ± 12^∗∗^
Sex: male (%)	7 (63%)	32 (63%)	71 (63%)
BMI	29 ± 9	32 ± 8	33 ± 7
Frailty score	3.4 ± 1.3	2.9 ± 1.0	2.8 ± 0.9^∗^
HLOS	33 (20–52)	23 (12–37)	19 (11–33)^∗^
ICULOS	26 (14–39)	17 (6–32)	11 (4–23)^∗^
Hypertension [*n* (%)]	4 (36%)	23 (45%)	48 (43%)
Diabetes mellitus	3 (27%)	9 (18%)	28 (25%)
COPD	3 (27%)	3 (9%)	0
VTE	0	3 (9%)	8 (7%)

^∗^ *p* < 0.05 compared with current vs former smokers.

^∗∗^ *p* < 0.05 compared with current vs never smokers.

## Data Availability

The datasets generated and/or analysed during the current study are not publicly available due to ethical restrictions but are available from the corresponding author on reasonable request.
